# Dietary Flavanols Modulate the Transcription of Genes Associated with Cardiovascular Pathology without Changes in Their DNA Methylation State

**DOI:** 10.1371/journal.pone.0095527

**Published:** 2014-04-24

**Authors:** Dragan Milenkovic, Wim Vanden Berghe, Céline Boby, Christine Leroux, Ken Declerck, Katarzyna Szarc vel Szic, Karen Heyninck, Kris Laukens, Martin Bizet, Matthieu Defrance, Sarah Dedeurwaerder, Emilie Calonne, Francois Fuks, Guy Haegeman, Guido R. M. M. Haenen, Aalt Bast, Antje R. Weseler

**Affiliations:** 1 INRA, UMR 1019, UNH, CRNH Auvergne, Clermont-Ferrand; Clermont Université, Université d'Auvergne, Unité de Nutrition Humaine, Clermont-Ferrand, France; 2 Laboratory of Eukaryotic Gene Expression and Signal Transduction LEGEST, University of Gent, Gent, Belgium; 3 PPES, Department of Biomedical Sciences, University of Antwerp (UA), Wilrijk, Belgium; 4 INRA, UMR1213 Herbivores, Plate-Forme d'Exploration du Métabolisme, Saint-Genès-Champanelle, France; 5 Department of Mathematics and Computer Science, University of Antwerp, Antwerp, Belgium; 6 Biomedical Informatics Research Center Antwerp (Biomina), University of Antwerp/Antwerp University Hospital, Edegem, Belgium; 7 Laboratory of Cancer Epigenetics, Free University of Brussels, Brussels, Belgium; 8 Department of Toxicology, Maastricht University, MD Maastricht, Netherlands; Queen's University Belfast, United Kingdom

## Abstract

**Background:**

In a recent intervention study, the daily supplementation with 200 mg monomeric and oligomeric flavanols (MOF) from grape seeds for 8 weeks revealed a vascular health benefit in male smokers. The objective of the present study was to determine the impact of MOF consumption on the gene expression profile of leukocytes and to assess changes in DNA methylation.

**Methodology/Principal Findings:**

Gene expression profiles were determined using whole genome microarrays (Agilent) and DNA methylation was assessed using HumanMethylation450 BeadChips (Illumina). MOF significantly modulated the expression of 864 genes. The majority of the affected genes are involved in chemotaxis, cell adhesion, cell infiltration or cytoskeleton organisation, suggesting lower immune cell adhesion to endothelial cells. This was corroborated by *in vitro* experiments showing that MOF exposure of monocytes attenuates their adhesion to TNF-α-stimulated endothelial cells. Nuclear factor kappa B (NF-κB) reporter gene assays confirmed that MOF decrease the activity of NF-κB. Strong inter-individual variability in the leukocytes' DNA methylation was observed. As a consequence, on group level, changes due to MOF supplementation could not be found.

**Conclusion:**

Our study revealed that an 8 week daily supplementation with 200 mg MOF modulates the expression of genes associated with cardiovascular disease pathways without major changes of their DNA methylation state. However, strong inter-individual variation in leukocyte DNA methylation may obscure the subtle epigenetic response to dietary flavanols. Despite the lack of significant changes in DNA methylation, the modulation of gene expression appears to contribute to the observed vascular health effect of MOF in humans.

## Introduction

A double-blind, randomized, placebo-controlled intervention study recently revealed, that the daily consumption of monomeric and oligomeric flavanols (MOF) derived from seeds of grapes (*Vitis vinifera* L.) for 8 weeks accomplish a vascular health benefit in male smokers [1]. This potential cardiovascular health benefit is in agreement with a recent study that showed an association between the intake of flavanols and reduced risk of coronary heart diseases [2]. However, comprehensive understanding of the biological activity of flavanols including their mechanisms of action in humans is still enigmatic [3].

Over the past years, *in vitro* and *in vivo* studies have shown that polyphenols modulate the activity of cell signalling proteins, transcription factors and consequently the expression of both, mRNA and miRNA [4]. For example, in animal models (mice and rats), supplementation of a diet with quercetin, naringenin or curcumin at nutritionally relevant doses altered the expression of hundreds of genes in lung, aorta or liver [5], [6], [7]. *In vitro*, polyphenols impact the expression of genes in various cell types. It has been demonstrated that flavanone metabolites at physiological concentrations can modulate the expression of genes involved in inflammation in tumor necrosis factor (TNF)-α-stimulated human umbilical vein endothelial cells (HUVECs) [8]. Exposure of human monocytes to green tea polyphenols affected the expression of genes related to atherosclerosis development, such as *CD36*, *LXR-α*, *MYC* or *LDL-R* [9]. In human hepatocytes polyphenols from red grape juice were able to counteract the LDL-induced changes in gene expression of the LDL receptor, hydroxymethylglutaryl-CoA (HMG-CoA) reductase and the transcription factor sterol regulatory element-binding protein (SREBP)-1, a key regulator of lipid homeostasis. Also genes such as *ICAM1*, *MCP1*, *IL6* or *IL1β* of which expression is controlled by nuclear factor kappa B (NF-κB) and of which transcriptional activation is critical in a number of pathologies, including cardiovascular diseases, are differentially expressed in the presence of polyphenols [10], [11]. However, only a few studies have been published up to now describing the genomic impact of polyphenols and polyphenol-rich foods in humans. It has been observed that the daily intake of 150 mg quercetin for 2 weeks significantly affected the expression of 788 genes in CD14-positive monocytes, genes related to immune system, apoptosis or cell signalling pathways [12]. Consumption of orange juice rich in hesperidin or hesperidin alone for 4 weeks by healthy, middle-aged, moderately overweight men significantly affected over 3000 and 1800 genes respectively. These genes were involved in the regulation of chemotaxis, cell adhesion, lipid transport and their expression profile could be considered as anti-inflammatory and anti-atherogenic [13]. More recently, in a double-bind, randomized cross-over trial in menopausal women isoflavones affected in peripheral blood mononuclear cells the expression of 357 genes which are involved in inflammation, oxidative phosphorylation and cell cycle [14].

In addition to the impact of polyphenols on the expression of genes, recent data suggest that polyphenols could also modulate DNA methylation at multiple levels [15], [16]. Furthermore, different lifestyle factors such as diet and smoking have been associated with DNA methylation changes in cancer and chronic inflammation related diseases such as metabolic syndrome, diabetes or cardiovascular diseases [17], [18], [19], [20], [21]. Whether diet specific epigenetic changes can also be detected in circulating blood leukocytes or contribute to disease progression is a hot research topic [22], [23], [24], [25]. The correlation of genome-wide data on DNA methylation and gene expression unveiled that methylation of promoter regions is frequently associated with transcriptional repression of genes being under control of this promoter [26], [27]. On the other hand it was also observed that this simple relation is not universally applicable but rather depends on genes, cell types, tissues and genetic variants [28], [29], [30]. The majority of these findings are derived from *in vitro* experiments with cell cultures. However, data that directly link DNA methylation changes with alterations in gene expression in humans are scarce. A first randomized controlled clinical study recently reported that the controlled intake of a cocoa extract by humans at cardiovascular risk is able to affect both global DNA methylation of white blood cells, as well as the expression of individual genes involved in the regulation of DNA methylation [25]. Whether transcriptional changes induced by the regular consumption of dietary polyphenols on a whole genome level can be related to changes of the methylome in white blood cells has yet not been investigated.

Taken together, it can be hypothesized that dietary flavanols are able to modulate the expression of genes being associated with CVD pathomechanisms via changes in the DNA methylation pattern of these genes. The present study aimed at investigating the impact of an 8 weeks controlled dietary intervention with MOF in smokers, *i.e.* humans with an increased risk of CVD, on genome-wide changes in leukocytes' gene expression and relate them to changes in DNA methylation.

## Materials and Methods

### Clinical trial

Details on the design, protocol and conduction of the clinical study are provided elsewhere [1]. In brief, non-obese, healthy male smokers in an age between 30 and 60 years and smoking 10 and more cigarettes per day for at least 5 years were included in the trial. The study was approved by the Medical Ethical Committee of the Maastricht University and Academic Hospital Maastricht, The Netherlands (ClinicalTrials.gov, NCT00742287) and conducted according to the World Medical Association Declaration of Helsinki of 1975, revision 2008. All subjects provided their written informed consent to participate in this study. For the present investigations blood samples of 13 men were available before and after an intervention of 8 weeks with a daily intake of two capsules containing 100 mg MOF from *Vitis vinifera* L. seeds (MASQUELIER's Original OPCs, INC BV, Loosdrecht, The Netherlands). The composition of the MOF capsules is specified in [1]. Basically, the intake of 2 capsules per day provided each subject with 51.3 mg total catechins ((+)−catechin, (−)-epicatechin and (−)−epicatechin-3-O-gallate), 55 mg total flavanol dimers (proanthocyanidin B1, B2, B3 and B4) and 93.8 mg total tri-, tetra- and pentameric proanthocyanidins. During the intervention period subjects maintained their usual smoking, dietary and lifestyle habits, complied with the regular intake of the capsules and did not experience any severe side effects.

**Figure 1:**
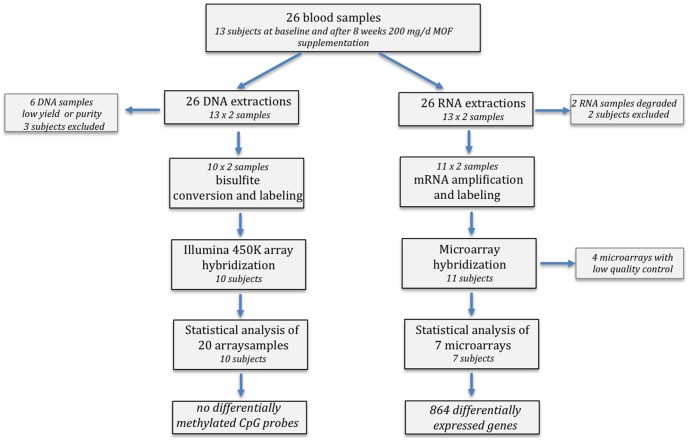
Flow chart of the sample analyses in the study.

The anthropometric and clinical characteristics of the volunteers from which the blood samples were derived for the analysis of gene expression and DNA methylation are given in **table 1**. An overview of the subsequent data analysis strategy and selection of RNA and DNA samples included in our data analyses is summarized in the flow chart (**figure 1**). Differential blood counts [1] of the samples for which the whole genome methylation state could be determined did not indicate any significant changes in the total number of leukocytes and the various subpopulations during the 8 weeks intervention period (**table 2**).

**Table 1:** Anthropometric and clinical characteristics of the male volunteers.

	Men (n = 13)	Clinical reference values
Age, y	48 (30–58)	n.a.
BMI, kg/m	25 (18–28)	<30
Years of smoking	28 (14–42)	n.a.
Cigarettes/day	16 (10–25)	n.a.
Pack years	19 (7–52)	n.a.
SBP, mmHg	115 (103–124)	<140
DBP, mmHg	74 (60–85)	<90
Heart rate, beats/min	55 (50–66)	60–100
tChol, mmol/L	5.7 (4.0–7.1)	≤5.0
LDL, mmol/L	3.9 (2.5–5.5)	≤2.5
HDL, mmol/L	1.2 (0.8–2.0)	0.9–1.7
TG, mmol/L	1.2 (0.9–4.0)	<2.0
CRP, mg/L	1.7 (0.6–5.6)	<10
Fibrinogen, g/L	3.4 (2.4–4.8)	2.0–4.0

Values are median (range); BMI, body mass index; CRP, C-reactive protein; DBP, diastolic blood pressure; HDL, high-density lipoprotein; LDL, low-density lipoprotein; n.a., not applicable; SBP, systolic blood pressure; tChol, total cholesterol; TG, triglycerides.

Clinical reference values are Dutch reference values “clinical chemistry” (www.fk.cvz.nl) and from the Dutch General Practitioner Guidelines (https://www.nhg.org/standaarden/volledig/cardiovasculair-risicomanagement [68]).

**Table 2:** Total and differential leukocytes count of the samples used for DNA-methylation analysis.

	Baseline (n = 10)	8 weeks (n = 10)	P value	Clinical reference values
Leukocytes, 10^9^/L	8±3	8±2	0.620	4–10×10^9^/L
Neutrophils, 10^9^/L	4 (3.2–11.7)	4.2 (2.8–6.7)	0.912	2–7.2×10^9^/L
Lymphocytes, 10^9^/L	2.3±0.6	2.3±0.6	0.868	1.0–4.0×10^9^/L
Monocytes, 10^9^/L	0.6±0.2	0.6±0.2	0.403	0.15–0.9×10^9^/L
Eosinophil granulocytes, 10^9^/L	0.3±0.1	0.3±0.1	0.841	0–0.5×10^9^/L
Basophil granulocytes, 10^9^/L	0.04±0.03	0.04±0.03	0.853	0–0.15×10^9^/L

Values are median (range) in case of non-normally distributed data or mean ± standard deviation (SD) in case of normally distributed data. Statistically significant differences between baseline and the end of the 8 week intervention were tested with two-tailed independent samples Student's t-test in case of normally distributed data or with the Mann-Whitney U-test in case of non-normally distributed data. P<0.05 is set as level of significance. Clinical reference values are Dutch reference values “clinical chemistry” (http://www.fk.cvz.nl).

### Sample collection, total RNA and genomic DNA extraction

At baseline and after 8 weeks of MOF supplementation freshly collected blood was added to RNALater (Ambion, Austin, TX, USA) and stored at −80°C until further extraction of RNA or genomic DNA (gDNA). Total RNA was isolated from blood by means of the RiboPure-Blood kit (Ambion, Austin, TX, USA) in accordance with the manufacturer's instructions. RNA purity and concentrations were determined by UV-VIS spectrophotometry (NanoDrop, Thermo Fisher Scientific Inc, Wilmington, DE, USA), and confirmed using the Agilent Bioanalyzer (Agilent Technologies Inc., Santa Clara, CA, USA). gDNA from EDTA-treated blood was extracted by a DNeasy blood & tissue kit (Qiagen, Courtaboeuf, France). DNA purity and concentrations were determined by UV-VIS spectrophotometry (NanoDrop, Thermo Fisher Scientific Inc, Wilmington, DE, USA) and stored at −80°C until further use.

### Microarray analysis

Fifty ng of total RNA extracted from each sample were amplified and fluorescently labelled to produce Cy5 or Cy3 cRNA using the Low Input Quick Amp Labeling two-color kit (Agilent, USA) in the presence of spike-in two colours control. After purification, 825 ng of labelled cRNA/sample were hybridized onto G4845A Human GE 4x44K v2 microarrays (Agilent, USA) according to the manufacturer's instructions. The G4845A Human GE 4x44K v2 microarray contains 27,958 Entrez Gene RNAs sequences. After hybridization, microarrays were scanned with Agilent G2505 scanner (Agilent, USA) and data were extracted with Feature Extraction software (Agilent, USA) using Lowess normalization. Statistical analyses were performed using the free R 2.1 software (http://www.r-project.org) after controlling the variance of each gene. Data were analyzed with a Student's t test to detect differentially expressed genes and the probability values were adjusted using the Benjamini-Hochberg correction for multiple testing at 0.1 to eliminate false positives. Genes selected by these criteria are referred to as the “differentially expressed genes”. Microarray data have been deposited in accordance to the MIAME guidelines in the public Gene Expression Omnibus (GEO) database (http://www.ncbi.nlm.nih.gov/geo/), accession number GSE54325 and GSE54643.

### Biological interpretation

Gene ontology (GO) annotations of biological processes for differentially expressed genes were conducted using MetaCore (https://portal.genego.com) and BioMart data mining tool of Ensembl database (http://www.ensembl.org/biomart/). To extract maximum biological information of differentially expressed genes, together with GO, genes were also classified according to their role(s) in cellular or metabolic pathways using Kyoto Encyclopedia of Genes and Genomes (KEGG) (http://www.genome.jp/kegg/pathway.html) and MetaCore (https://portal.genego.com) databases. Potential transcription factors involved in the regulation of differentially expressed genes were searched using network algorithms for transcription factors development in MetaCore software.

### Human monocyte to endothelial cell adhesion in vitro assay

Primary human umbilical vein endothelial cells (HUVECs; Lonza, Walkersville, MD, USA) were used at passage 5 and were cultured in a phenol-red-free endothelial growth medium (EGM) supplemented with 2% fetal bovine serum (FBS), 0.4% fibroblast growth factor, 0.1% vascular endothelial growth factor, 0.1% heparin, 0.1% insulin-like growth factor, 0.1% ascorbic acid, 0.1% epidermal growth factor, and 0.04% hydrocortisone (all from Lonza). Experiments were performed in 24 well plates (Becton Dickinson, Le Pont de Claix-Cedex, France). A human monocytic cell line (THP-1) (ATCC, Manassas, VA, USA) was cultured in RPMI 1640 medium (Pan Biotech, Aidenbach, Germany) supplemented with 2% FBS (Sigma, Saint Quentin Fallavier, France). Both cultures were maintained at 37°C and 5% CO2.

For cell adhesion assays, when HUVECs reached 60–70% confluence, monocytes were exposed for 24 h to MOF dissolved in mQ to a final concentration of 0.05 µg/ml, 0.5 µg/ml and 5 µg/ml. After 24 h, the confluent HUVEC monolayer was stimulated for 4 h with TNF-α at 0.1 ng/ml or PBS/BSA (0.01‰, negative control). Following TNF-α stimulation, 50 µl of a 5×10^6^/ml THP-1 suspension with or without pre-exposure to MOF, was added to each well and cells were further incubated for 1 h. Non-adhering THP-1 cells were rinsed away with PBS. The remaining cells were fixed and stained with crystal violet 0.5% (m/V) in ethanol (Sigma, France). The number of attached monocytes was counted for each well in three random microscopic fields defined by an eyepiece. Triplicates for each condition were performed in three independent experiments.

### NF-κB reporter gene in vitro assay

A human monocytic cell line stably transfected with a luciferase reporter containing three NF-κB-binding sites (U937-3xB-LUC cells) was used to investigate the effects of MOF on NF-κB mediated gene expression [31]. Cells were cultured in RPMI-1640 medium supplemented with L-glutamine (2 mM), penicillin (50 U/ml), streptomycin (50 mg/ml), hygromycin (75 µg/ml) and 10% fetal bovine serum at 37°C and 5% CO_2_. For experiments cells were transferred to complete medium without antibiotics in 24 well plates. For lipopolysaccharide (LPS)-induced NF-κB activity, monocytes were *in vitro* differentiated to macrophages by 24 h PMA treatment (10 ng/ml) after which the medium was replaced by complete medium without PMA. Subsequently, cells were incubated with MOF (0.05–5 µg/ml) or vehicle control (mQ) for 30–60 min, followed by the addition of LPS (1 µg/ml) and an additional incubation of 4–6 h prior to measuring luciferase reporter-gene expression. In another experimental set-up monocytes were differentiated to macrophages by 24 h PMA (10 ng/ml) treatment in the presence or absence of MOF (5 µg/ml). After replacement of the medium, macrophages were stimulated with LPS (1 µg/ml) for 6 h before luciferase reporter-gene expression was determined. Cell lysis and luciferase (luc) assays were carried out according to the protocol of Promega Corp. (Madison, WI, USA). The preparation of luciferase (luc) reagent was described previously [32]. Cell viability was determined by trypan blue exclusion.

### DNA-methylation arrays

Bisulphite converted DNA from the subjects was hybridized to the Illumina HumanMethylation450 BeadChip arrays. For each sample, 1 µg of genomic DNA was bisulfite-converted using an EZ DNA methylation Kit (ZYMO research) according to the manufacturer's recommendations. Converted genomic DNA was eluted in 22 µl of elution buffer. DNA methylation level was measured using the Illumina Infinium HD Methylation Assay (Illumina) according to the manufacturer's instructions. Briefly, 4 µg of bisulfite-converted DNA was isothermally amplified overnight (20–24 h) and fragmented enzymatically. Precipitated DNA was resuspended in hybridization buffer and dispensed onto the Infinium HumanMethylation450 BeadChips (12 samples/chip) using a Freedom EVO robot (Tecan). The hybridization procedure was performed at 48°C overnight (16–20 h) using an Illumina Hybridization oven. After hybridization, free DNA was washed away and the BeadChips were processed through a single nucleotide extension followed by immunohistochemistry staining using a Freedom EVO robot (Tecan). Finally, the BeadChips were imaged using an Illumina iScan. Illumina data have been deposited in accordance to the MIAME guidelines in the public Gene Expression Omnibus (GEO) database (http://www.ncbi.nlm.nih.gov/geo/), accession number GSE 54690.

### Illumina data analysis

Raw Illumina data were filtered by Genomestudio software and R packages Bioconductor Minfi and Rnbeads to remove low quality data using a detection P-value threshold of 0.05. Cross-reactive probes (*i.e*. targeting several genomic locations) and probes containing SNPs were filtered out using the extended annotation provided by Price *et al* [33] (see [34] for a detailed description). β-values were computed using the formula β-value = M/[U+M] where M and U are the raw “methylated” and “unmethylated” signals, respectively. Beta-values were corrected for the type I and type II bias using the peak-based correction [34].

### DNA CpG pyrosequencing

Bisulfite modification of 1 µg of gDNA was performed using the EpiTect Fast DNA Bisulfite kit (Qiagen) per manufacturer's protocol. Forward, reverse, and sequencing primers were designed using the Pyromark Assay Design Software (Qiagen Inc., Valencia, CA). Bisulfite converted DNA was diluted 1∶50 with water. A touchdown PCR program was used to amplify the DNA using the HotStarTaq kit (Qiagen Inc., Valencia, CA). PCR cycling conditions were as follows: denaturation at 95°C for 30 seconds, annealing (temperature varied depending upon primer and replication cycle) for 30 seconds, extension at 72°C for 1 minute for 45 cycles followed by a final extension at 72°C for 10 minutes. Samples were then stored at 4°C. The following primer sets were used for DNA CpG pyrosequencing: *CCR6* forward primer TTGTGTTTGGAGTAGTGTTTAGT, reverse primer Biotin-CCTAAATCTTCCACCTCCTCTTTATACCAC, sequencing primer TGTTTGGAGTAGTGTTTAGTA; *MAFB* forward primer AGGGAATTGAGGGAGGAGAG, reverse primer Biotin-ACCCACCCCCCTACCCTACAAA, sequencing primer GTGATTTGGTTTAGAGGT; *PCDHB4* forward primer AGATTTTTGGTTAGATGGGTTATAAAG, reverse primer Biotin-ATTCATCCAAACAAACAAATATTAACC, sequencing primer ATGGGTTATAAAGGAGT. CpG methylation percentages were calculated using the height of the T and C peaks at the methylation site and applying the formula (C/C+T)×100 as implemented in the Pyromark CpG software (version 1.0.11).

### Statistics

The effects of MOF on monocyte-endothelial cell adhesion were analysed by means of two-way analysis of variance (ANOVA) followed by Tukey's multiple comparisons post-hoc tests.

To examine the effects of MOF on NF-κB reporter gene activity or DNA methylation either one-way or two-way ANOVA was used as indicated. Significant differences were identified using Bonferroni's or Tukey's multiple comparisons post-hoc tests (reporter gene assay data) or Bonferroni's post-hoc test (DNA methylation), as indicated. For correlation analysis, the Pearson coefficient was calculated. The level of statistical significance was set to P<0.05 for all analyses. Statistical analyses were performed with GraphPad Prism 6.0 (GraphPad, La Jolla, CA, USA).

## Results

### Baseline characteristics of the included male smokers

**Table 1** shows that the median BMI of the included volunteers was at the upper limit of normal, and none of subjects was obese, i.e. having a BMI ≥30 kg/m^2^. All volunteers were normotensive and had elevated serum LDL-cholesterol levels. In addition, the majority of these persons (n = 10) had total cholesterol concentrations above 5 mmol/l serum. The white blood cell composition did not significantly fluctuate during the 8-week intervention period (**table 2**).

### Nutrigenomic analysis of transcriptional effects of MOF supplementation in blood leukocytes

RNA samples from the blood cells of 13 volunteers at baseline and after 8 weeks of MOF consumption was used for the microarray study (**figure 1**). The concentration of total RNA per blood sample was 47.5 ng in average. However, 2 RNA extractions were eliminated due to low RNA yields. Following hybridization of microarrays, 4 samples did not meet quality control conditions and were eliminated. After paired statistical analysis followed by false positive correction of the data, modification in the gene expression profile was observed following 8 weeks consumption of MOF. Statistical analyses revealed 864 differentially expressed genes (p>0.05 and corrected p<0.1) (**table S1**). Out of the 864 genes, 445 were identified as down-regulated and 419 as up-regulated, with expression changes varying from −1.32 to 1.72.

**Figure 2:**
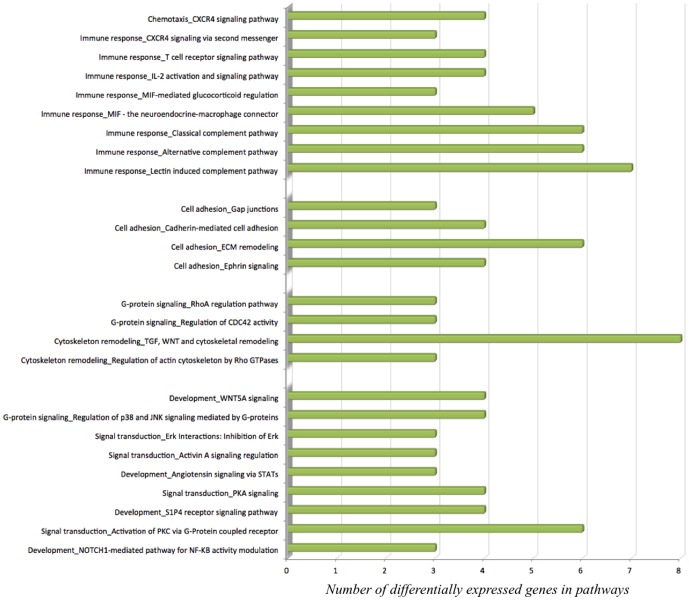
Significant pathways (MetaCore MetaCore Bioinformatics software from Thomson Reuters,https://portal.genego.com/) of the gene expression profiles of leukocytes after consumption of monomeric and oligomeric flavanols (MOF) derived from grape seeds involved in immunity, cell signalling and cell adhesion.

**Figure 3:**
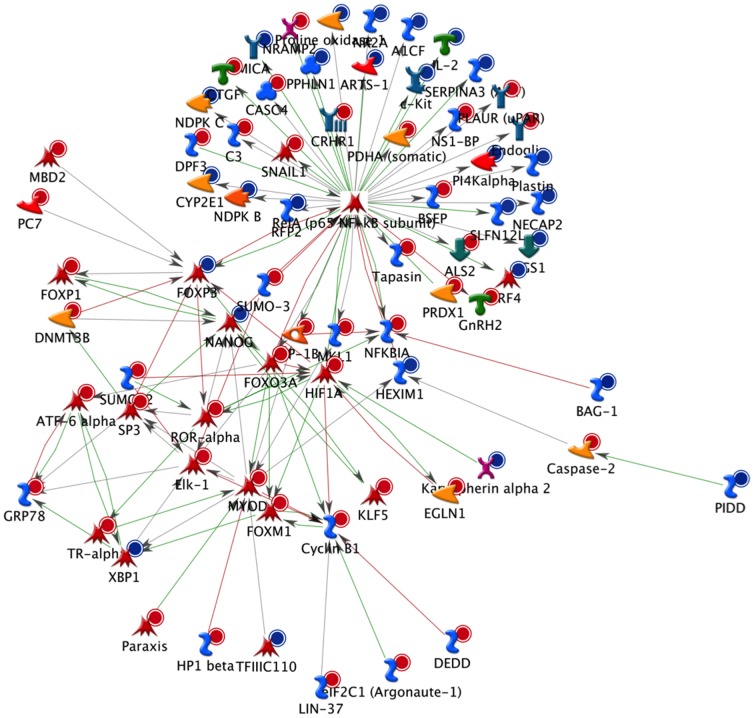
An *NF-κB* biological network based on the differentially expressed genes in circulatory cells obtained from MetaCoreTM network software and the “Analyze” network algorithms. Dots in the right corner of a gene indicate differential expression, blue for down-regulated genes and red for up-regulated genes.

To identify the biological function in which these genes are involved, gene ontology, pathway and gene network analyses were performed. Gene ontology revealed that differentially expressed genes are involved in different over-represented cellular processes (**table S2**). Among these processes are those regulating macrophage-derived foam cell differentiation, chromatin organization, cell cycle, chemotaxis, regulation of cell-cell adhesion, regulation of metabolic processes, regulation of lipid storage and regulation of cytoskeleton organization. To further refine the biological functions in which differentially expressed genes are implicated, the genes were placed according to their role(s) in cellular or metabolic pathways. Pathway analyses of the differentially expressed genes using MetaCore software identified 59 significantly over-represented cellular pathways (**table S3**). Among these 59 pathways, 19 are involved in the regulation of inflammation, cell adhesion, cell cycle and cytoskeleton remodelling (**figure 2**). Pathway analysis using KEGG database also revealed pathways such as cell adhesion, MAPK signalling pathway, regulation of actin cytoskeleton, cell cycle, chemokine signalling pathway, focal adhesion and leukocyte transendothelial migration. Among the genes present in these pathways are *CXCL12*, *SCRIB*, *PDGFRL*, *FERMT3*, *ICAM1* or *VCAM1*. As for MetaCore, KEGG database pathways also indicated cell adhesion, chemotaxis or regulation of cellular cytoskeleton as significantly altered pathways upon MOF supplementation. Moreover, bioinformatic analyses were performed on the differentially expressed genes with the aim to identify transcription factors that could be modulated by MOF and which are potentially involved in the regulation of the expression of the identified genes. Among the identified transcription factors are *SP1*, *p53*, *AP1* and *NF-κB*. A network of genes centred to *NF-κB* and affected by the MOF supplementation is presented in **figure 3**.

### Effects of MOF on monocyte adhesion to human vascular endothelial cells in vitro

**Figure 4:**
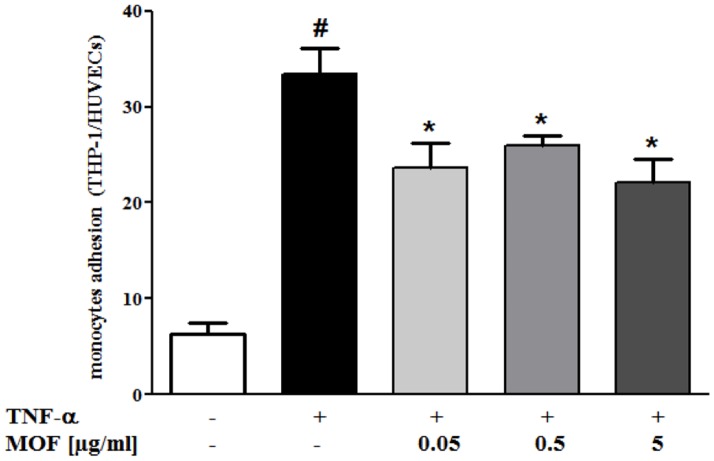
Effect of incubation of THP1 monocytes with MOF concentrations ranging from 0.05–5 µg/ml on adhesion to human umbilical vein endothelial cells (HUVEC). Monocyte adhesion is presented as a ratio of THP1 to HUVEC. Values are means of three independent experiments, with standard deviations represented by vertical bars. Data were analysed using two-way analysis of variance (ANOVA) and significant differences were identified using Tukey's multiple comparisons post-hoc test. Statistical significant difference was set to P<0.05 for all analyses, where * indicating significant different to TNF-α and # indicating significant different to negative control.

To substantiate the findings from the microarray analyses that exposure of circulating white blood cells to MOF modifies the expression of genes towards reduced immune cell-endothelial cell interaction, we performed *in vitro* monocyte to endothelial cell adhesion assays (**figure 4**). We observed that the number of monocytes adhering to HUVECs without TNF-α stimulation was low. Stimulation of the endothelial cells with 0.1 ng/ml TNF-α significantly induced monocyte adhesion to endothelial cells by a factor of 6. Interestingly, pre-exposition of the monocytes to different concentrations of MOF significantly reduced their adhesion to TNF-α-stimulated endothelial cells by 21% to 34% (**figure 4**). However, under these experimental conditions, we did not observe a dose-dependent inhibition.

### Effects of MOF on NF-κB activity in human monocytes/macrophages in vitro

**Figure 5:**
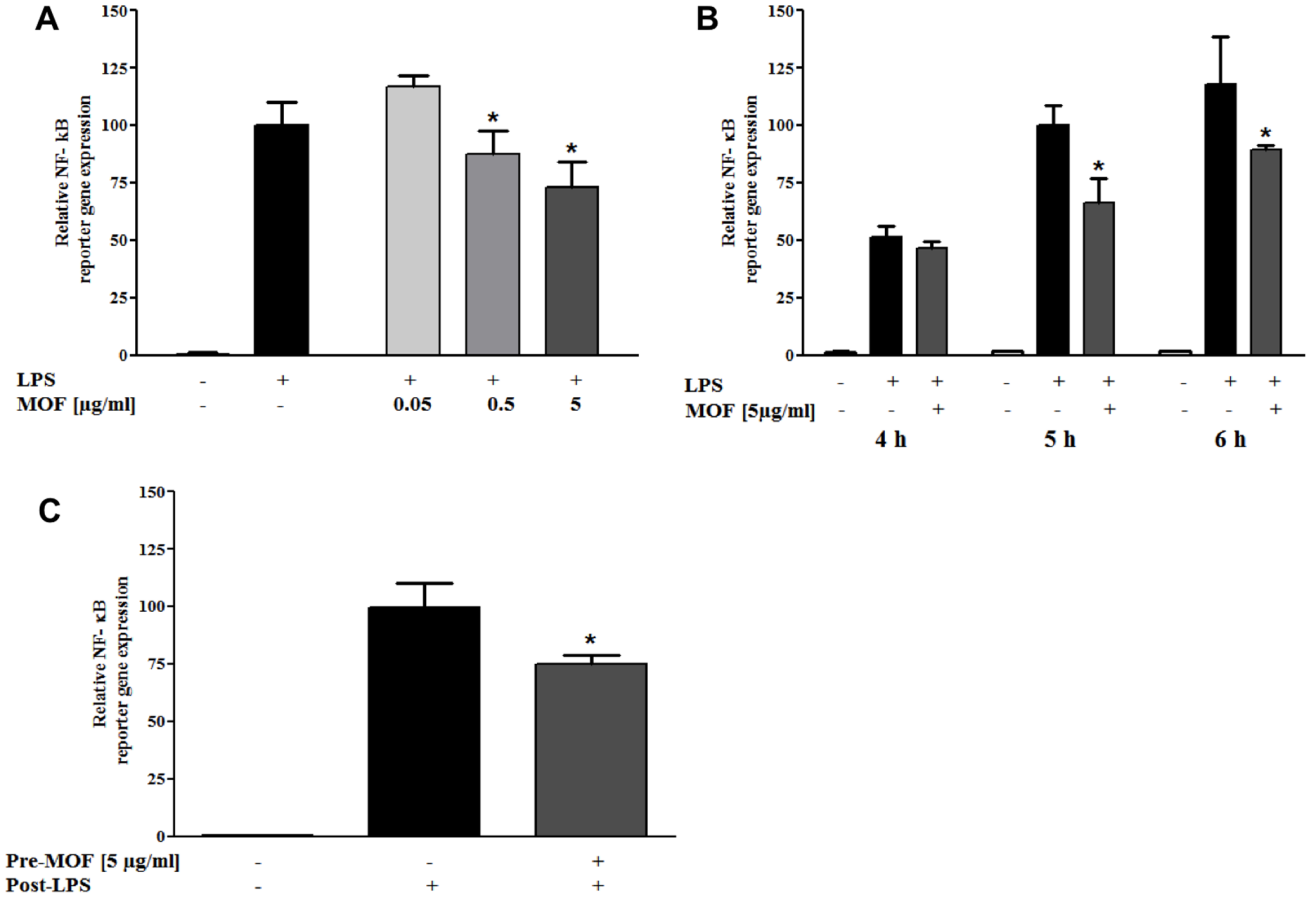
Effects of MOF on NF-κB mediated reporter gene expression. A) Dose-dependent inhibition of 5 h lipopolysaccharide (LPS)-induced (1 µg/ml) NF-κB dependent reporter gene transcription by 30 min. pre-incubation of *in vitro* differentiated U937 macrophages with MOF concentrations ranging from 0.05–5 µg/ml. One-way ANOVA was used to evaluate significant effects on reporter gene activity induced by MOF. Significant differences between single LPS treatment and LPS combination treatment with MOF concentrations ranging from 0.05–5 µg/ml were identified using Tuckey's multiple comparisons post-hoc test. B) NF-κB dependent reporter gene expression in *in vitro* differentiated U937 macrophages exposed for 4, 5 or 6 h to LPS (1 µg/ml), with or without 60 min. pre-incubation with 5 µg/ml MOF. Two-way ANOVA and Bonferroni's post-hoc test were used to determine significant time- and MOF concentration-dependent effects on LPS-induced reporter gene activity. C) U937-3xB-LUC monocytes were *in vitro* differentiated for 24 h with PMA (10 ng/ml) in the presence or absence of 5 µg/ml MOF. Upon replacement of the medium in the different settings, cells were exposed to LPS for 6 h. One-way ANOVA was used to examine significant MOF effects on reporter gene activity. Significant differences between single LPS treatment and LPS treatment following pretreatment with MOF were evaluated using Tuckey's multiple comparisons post-test. All values are means of three independent experiments, with standard deviations represented by vertical bars, * indicating statistically significant differences assessed as described accordingly (P<0.05).

To experimentally confirm the predicted impact of MOF on the activity of the transcription factor NF-κB, we performed NF-κB-specific reporter gene assays in the U937 human macrophage cell line. *In vitro* differentiated U937-3xB-LUC macrophages were left untreated or treated with different concentrations of MOF for 30 or 60 minutes, respectively (**figure 5 A and B**). Hereafter, cells were exposed to LPS (1 µg/ml) for 4, 5 or 6 h after which cells were lysed and analysed for luciferase reporter-gene expression. As presented in **figure 5**, a moderate but significant dose- (**figure 5 A**) and time-dependent (**figure 5 B**) reduction in NF-κB reporter gene activity could be observed upon combined treatment with LPS and MOF. Alternatively, to evaluate persistent “memory” effects of MOF on NF-κB-dependent gene expression, cells were either treated with PMA (10 ng/ml) alone or co-treated with 5 µg/ml MOF and PMA (10 ng/ml) during 24 h monocyte-macrophage differentiation. Twenty-four hours following replacement of the medium, cells were stimulated for 6 h with LPS (1 µg/ml). Cells differentiated in the presence of both, PMA and MOF, revealed a significant lower maximal NF-κB response upon subsequent LPS stimulation (**figure 5 C**).

### Intra- and inter-individual changes in genome-wide DNA methylation analysis of blood samples before and after MOF supplementation

**Figure 6:**
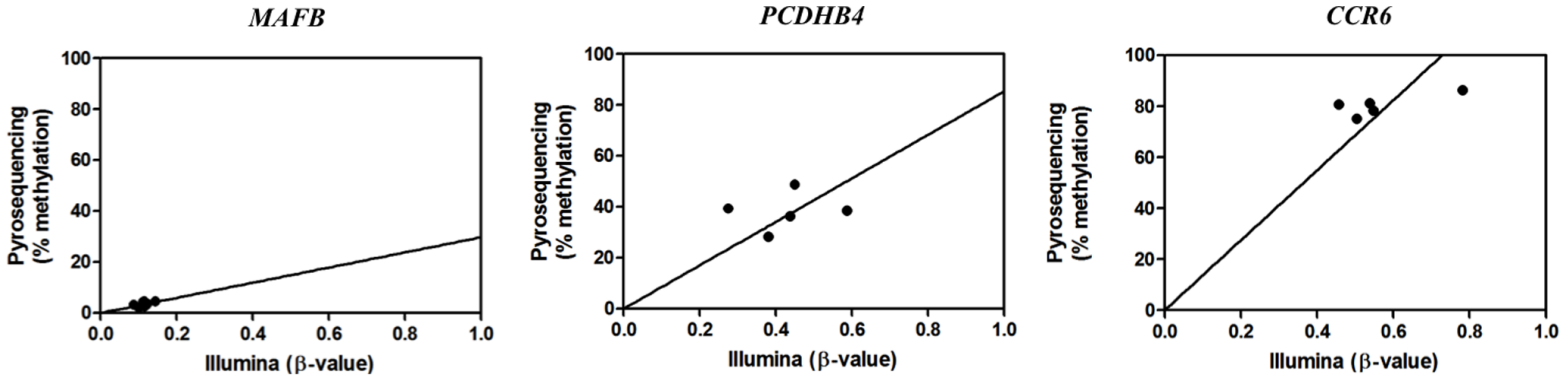
CpG pyrosequencing-based validation of Illumina 450K array results. DNA methylation of blood leukocyte gDNA of 5 volunteers was bisulfite treated and amplified by specific biotinylated primer sets for respectively a highly methylated CpG probe region of the *CCR6* gene, a medium methylated CpG probe region of the *PCDHB4* gene and a weakly methylated CpG probe region of the *MAFB* gene. DNA methylation intensities obtained from the different CpG probes by CpG pyrosequencing were plotted against the β-values (0<x<1, reflecting 0–100% DNA methylation) obtained with the Illumina 450K array platform. Curve fitting reveals significant correlation of results between both platforms.

**Figure 7:**
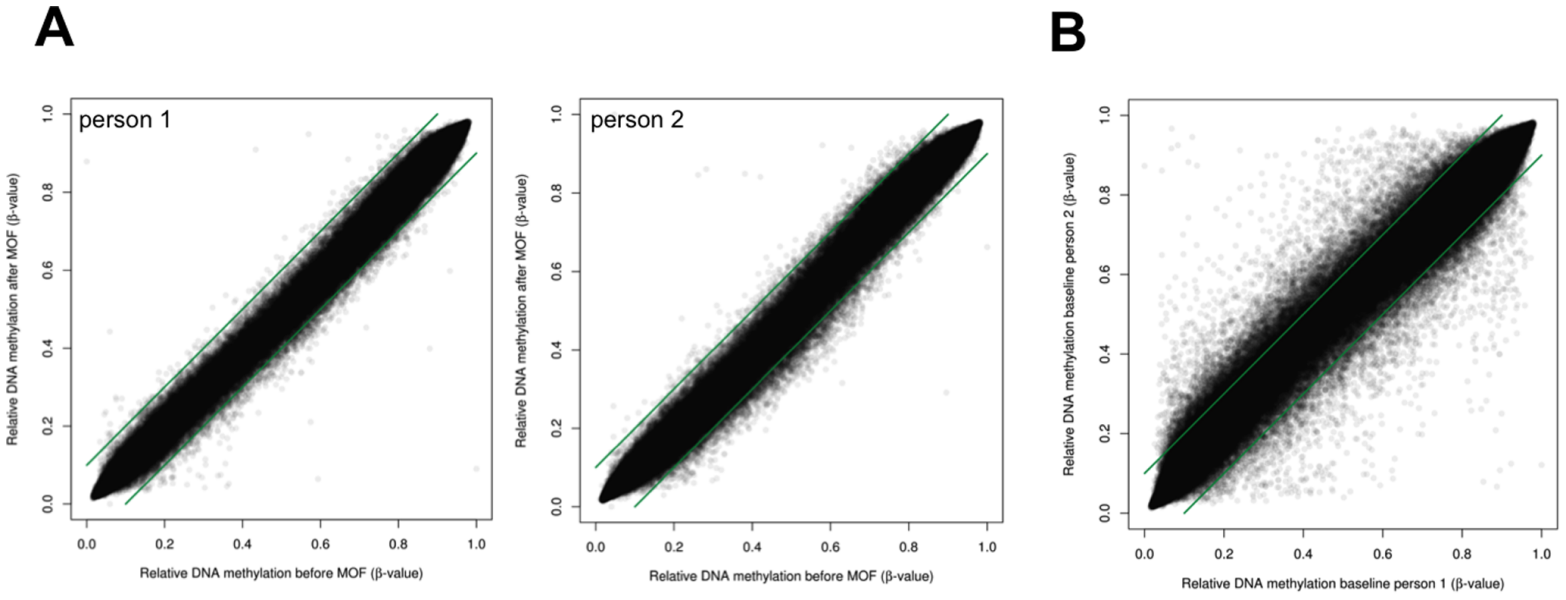
Intra-individual DNA methylation before and after MOF supplementation. Genome wide DNA methylation profiles of blood leukocyte gDNA of 10 individuals were profiled by Illumina 450K CpG array. A) DNA methylation intensities (β-values 0<x<1, reflecting 0–100% DNA methylation) of 450,000 CpG probes were plotted as dots along the X and Y axis to compare DNA methylation variation before and after 8 weeks MOF supplementation in 2 representative subjects. B) DNA methylation intensities (β-values 0<x<1, reflecting 0–100% DNA methylation) of 450,000 CpG probes were plotted as dots along the X and Y axis to compare DNA methylation variation at baseline between 2 representative subjects. Green lines represent cut-off values of 10% difference in β-values.

To evaluate whether transcriptional changes observed in the microarray experiment correspond with epigenetic changes in DNA methylation, gDNA from 10 volunteers at baseline and after 8 weeks of MOF consumption was analyzed for gene-specific DNA methylation changes by Illumina 450K bead array. 99% of the CpG probes resulted in reliable hybridisation signals (P<0.01). Low (β-value<0.2), medium (0.2<β<0.6) and highly methylated Illumina CpG probes (β>0.6) of respectively *MAFB*, *PCDHB4* and *CCR6* were selected for further validation by CpG pyrosequencing (**figure 6**). This confirmed good overall correlation between Illumina CpG array and CpG pyrosequencing based quantification of DNA methylation trends, although small shifts (under/overestimation) could be observed for some CpG probes on the Illumina platform. Upon comparison of β-values of 450K CpG probes at baseline and after 8 weeks of MOF consumption, CpG probes were selected which revealed minimal 10% increase or decrease (i.e. 0.1 difference in β-value) between both conditions in different individuals or at the group level. Intra-individual DNA methylation before and after MOF supplementation of 2 representative subjects is illustrated in **figure 7A**. Typically, approx. 0.03 to 3% of the 450K CpG probes changed in different individuals during the 8 weeks MOF supplementation (data not shown). However, taking into account that experimental variation of sample replicates in the Illumina array may already result in false positive changes in 1% of the 450K CpG probes (P<0.01), no statistical significant DNA methylation changes could be associated with the MOF supplementation due to the small sample size (n = 10) and lack of replicate measurements.

Furthermore, upon comparing β-values of 450K CpG probes at baseline between subjects, we observed a remarkable epigenetic variation, since up to 10% of the CpG probes revealed more than 10% difference in DNA methylation (**figure 7B**). This difference could not be attributed to fluctuations in leukocytes composition and numbers as this was found to be consistent between individuals before and after the MOF intervention (**table 2**). Relative number of intra- versus inter-individual changes in CpG probe DNA methylation (>10%) for all individuals before/after MOF supplementation is summarized in table S4. Although cardiovascular risk factors such as smoking, hypertension, and high pulse seemed to change DNA methylation of various genes related to cell adhesion pathways, we could not associate statistical significant DNA methylation changes with specific lifestyle factors, due to the small cohort size (data not shown).

### Changes in DNA methylation of genes associated with cardiovascular pathways during 8 weeks MOF supplementation

**Figure 8:**
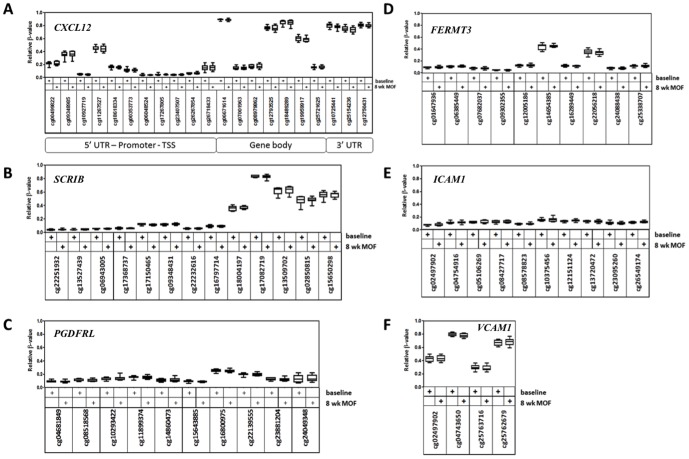
Gene-specific variation in DNA methylation before and after MOF supplementation. Box- and whisker plots represent DNA methylation as β-values (0<x<1, reflecting 0–100% DNA methylation) of 10 individuals before and after 8 weeks MOF supplementation for the following genes: *CXCL12* (A), *SCRIB* (B), *PDGFRL* (C), *FERMT3* (D), *ICAM1* (E) and *VCAM1* (F). In figure 8A CpG sites have been sorted according to their chromosome locus, including 5′-UTR, promoter (transcription start site region (TSS)), gene body and 3′-UTR region, as indicated in the figure. For figures 8B - F, only the 5′-UTR promoter region (TSS) has been included. No significant MOF effects on DNA methylation β-values could be observed by one-way ANOVA and Bonferroni's post-hoc test.

In order to examine the DNA methylation state of various regions of individual genes during the 8 weeks intervention we selected the following genes *CXCL12*, *SCRIB*, *PDGFRL*, *FERMT3*, *ICAM1* and *VCAM1*. These genes are involved in pathways related to cardiovascular diseases and have been shown to be differentially expressed during the 8 weeks MOF intake. Figure 8 shows the DNA methylation state of individual CpG in different gene regions before and after the MOF intervention. Interestingly, within an individual gene such as *CXCL12*, highly variable CpG methylation (low-medium-high methylation) could sometimes be observed for specific probes across 5′-UTR, promoter, gene body or 3′-UTR regions. No major DNA methylation changes (>10%) could be detected in any of the differentially expressed genes at group level, which is in line with our global DNA methylation analysis (Figure 7).

## Discussion

In animal models and *in vitro* studies, the potential beneficial effect of flavanols on the prevention of different diseases including cancer, neurodegenerative and cardiovascular diseases has been demonstrated [35]. This effect seems to be related to their capacity to modulate the activity of different enzymes, cell signalling proteins and expression of genes and proteins. A recent meta-analysis has revealed that grape seed extract appears to significantly lower systolic blood pressure and heart rate [36], and we have recently shown that consumption of grape seed-derived MOF presented an overall vascular health benefit [1]. The potential mechanism of action underlying these effects in humans is still largely unknown. In this study, we demonstrated that the consumption of MOF affected blood leukocytes gene expression without a significant modulation of changes in DNA methylation. The capacity of polyphenols to modulate gene expression profiles in circulating blood cells in humans has been described in a few clinical trials [12], [14]. These nutrigenomics studies have shown that hesperidin, quercetin, resveratrol or isoflavones can modulate the expression of 560 to over 4000 genes suggesting that the health benefits of polyphenols in humans is probably dependent on their genomic effects. Recently, the impact of the consumption of grape seed extract containing resveratrol during 6 and 12 months was studied on the expression of genes in peripheral blood mononuclear cells of type 2 diabetic and hypertensive medicated male patients [37]. Using a nutrigenomic approach, over 4000 genes have been differentially expressed upon the intervention which are involved in inflammatory cytokine-mediated processes, cell movement, cell signalling and cell trafficking. Compared to our study a higher number of differentially expressed genes was observed which might be due to differences in volunteers (patients versus healthy subjects), the composition of the investigational compounds, the doses used (300 and 600 mg/d versus 200 mg/d) and the length of the study (6 and 12 months versus 8 weeks). Comparison of the genes revealed only 145 common genes in both studies (**figure S1A**). However, the pathways identified from differentially expressed genes by means of KEGG database in our study coincide with those reported by Tomé-Carneiro et al [37] (**figure S1B**).

Bioinformatic analysis revealed that the differentially expressed genes are involved in the regulation of different cellular processes. The most overrepresented pathways are involved in chemotaxis, cell adhesion, immune response and cell cycle. Chemokines and adhesion molecules play important roles in leukocyte adhesion, trans-endothelial migration and activation [38], [39]. Among the genes involved in chemotaxis and leukocyte infiltration is *CXCL12* [40], which appeared to be down-regulated after 8 weeks MOF supplementation. Together with *CXCL12*, a decrease in the expression of *SCRIB*, a gene involved in chemotaxis and cell migration could be observed [41]. Chemotaxis of circulating blood cells is followed by their interaction with the vascular endothelium, which presents a first event in atheroma plaque formation, and requires the participation of different cell adhesion molecules. Over 30 genes coding for cell adhesion molecules have been identified in our study by means of gene ontology interpretation. Among these genes, we observed a down-regulation of the expression of *FERMT3*. This gene has been described as playing a significant role in cell-cell adhesion. The loss of expression of this gene resulted in abolishment of firm adhesion and arrest of neutrophils on activated endothelial cells *in vitro* and *in vivo*, without affecting selectin-mediated rolling [42]. Furthermore, the interactions between circulating blood cells and endothelial cells can also be regulated by interaction of leukocytes with platelets, a process that involves platelet-derived growth factors (PDGFs) [43]. In our study, we observed that a regular consumption of MOF significantly decreased the expression of PDGF receptors (*PDGFRL*), suggesting potential lower interactions of leukocytes with platelets and consequently endothelial cells. Taken together, the bioinformatic analyses showed that consumption of MOF modulate expression of genes involved in chemotaxis, adhesion and platelet adhesion, suggesting potential lower adhesion of circulating blood cells to the endothelium. In the objective to substantiate this finding on a cellular level, we exposed human monocytes to MOF and co-incubated them with vascular endothelial cells *in vitro*. In agreement with our nutrigenomic results, a significant decreased adhesion of monocytes pre-exposed to MOF could be observed at the endothelial cells compared to control. The potential of grape seed extract to decrease adhesion of monocytes (THP-1) to HUVECs has been reported recently, however at concentrations ranging from 50 to 100 µg/ml [44]. These concentrations are higher than the concentrations used in our study. Our results emphasize the capacity of grape seed–derived MOF to decrease the adhesion of immune cells to the vascular endothelium and potentially lower infiltration of these immune cells into the vascular wall, which is an initial step in atherosclerosis development. It has been reported that supplementation of the diet with catechin, a flavanol monomer present in MOF, results in lower atherosclerosis development in apolipoprotein E-deficient mice [45]. It could be postulated that the regular consumption of MOF could decrease blood cell infiltration into the vasculature and potentially protect against atherosclerotic lesions in humans.

Our bioinformatic data also revealed that over 30 differentially expressed genes are involved in different processes related to inflammation. The role of chronic inflammation in the promotion, initiation and development of chronic diseases, such as cancer [46], cardiovascular disease [47] or osteoporosis [48] has been described. In our study, among the differentially expressed genes involved in inflammatory processes is the gene coding for the pro-inflammatory cytokine *IL2*, of which the expression has been down-regulated by the MOF supplementation. Together with *IL2*, we also observed a decrease in the expression of a subunit of its receptor, the *IL2RB* gene. These inflammation-related genes, as well as some of the cell adhesion molecules, are regulated by the transcription factor NF-κB. Bioinformatic analyses of the nutrigenomic data identified several transcription factors potentially involved in the regulation of the expression of differentially expressed genes, among which is *NF-κB*. This suggests that NF-κB is a potential target by which MOF exert their anti-inflammatory effects in circulating blood cells. Interestingly, our nutrigenomic data also revealed an increase in the expression of the gene coding for *NFKBIA*, the endogenous inhibitor of NF-κB. A previous *in vivo* study in an atherosclerotic ApoE mouse model identified NF-κB as a major upstream regulator in leukocyte adhesion and transendothelial migration through the vascular endothelium [7]. Moreover, NF-κB activity in peripheral blood mononuclear cells (PBMCs) from smokers has been found to be significantly higher than in PBMCs from non-smokers [49]. In order to corroborate a direct effect of MOF on NF-κB we performed NF-κB reporter gene studies in an inflammatory human monocyte/macrophage cell model. The experiments revealed a dose- and time-dependent repression of NF-κB luciferase expression in the presence of MOF. As such, MOF may trigger synergistic cardio-protective effects by reducing NF-κB activity in both, monocytes and endothelial cells, resulting in attenuation of cell adhesion and transendothelial migration programs. Taken together, the nutrigenomic data elucidated numerous genes which are modulated by an 8 weeks supplementation with MOF in blood cells. The affected genes are involved in different cellular processes that regulate interactions with the endothelium and inflammation. We also found that regular consumption of MOF alters gene expression to a potentially protective cardiovascular profile. These results further unravel the molecular mechanisms of the vascular health benefits provided by dietary flavanols in humans.

Although DNA methylation effects have been described in peripheral leukocytes of humans at cardiovascular risk upon consumption of dietary polyphenols and cruciferous vegetables [25], we were unable to identify common DNA methylation changes in our group of subjects after 8 weeks MOF supplementation. One of the main reasons for this is the high inter-individual basal variation in DNA methylation in the recruited smokers. Of special note, tobacco smoke is known to trigger genome-wide changes in DNA methylation and may mask diet-induced effects of relatively short (i.e. compared to lifespan) intervention studies [50], [51], [52]. In addition to smoking, age or clinical parameters (blood pressure, pulse, LDL/HDL ratio, plasma CRP, BMI, serum cholesterol) may introduce variation in genome-wide DNA methylation patterns [50], [51], [52], [53], [54], [55], [56], [57], [58], [59]. Considering the stochastic nature of DNA methylation changes, we cannot exclude that a relative short diet intervention (8 weeks) is not sufficient to trigger a 10% change in DNA methylation (our cut-off criterion for differential methylation) as compared to DNA methylation changes following many years (>15 y) of heavy smoking, hypertension and/or aging. Indeed, minor diet-induced DNA methylation changes of 3–6% of the *NR3C1* gene have been linked to hyperhomocysteinemia in cardiovascular disease [60]. In a soy diet study in monkeys, it was observed that diet-specific DNA methylation changes may vary between the tissue: most pronounced DNA methylation effects were found in liver and muscle, whereas blood remained unaffected [61], [62]. As such, blood samples may not represent the most sensitive tissue to evaluate specific nutritional epigenetic changes. Analyses may be further complicated by different subtypes of blood cells [63], [64], [65]. Alternatively, timing of dietary exposure may be another critical parameter, as prenatal exposure to genistein was found to exert lifelong alterations in gene expression and DNA methylation of hematopoietic cells [66], [67].

In conclusion, our study revealed that a daily supplementation with 200 mg MOF over 8 weeks modulates the expression of genes associated with cardiovascular disease pathways without major specific changes of their DNA methylation state. A strong inter-individual variation in leukocyte DNA methylation may conceal the subtle epigenetic response to dietary flavanols. This calls for studies with larger sample sizes to reveal an epigenetic role of flavanols in cardioprotection. Despite the lack of significant changes in DNA methylation, we could demonstrate that the modulation of gene expression appears to contribute to the vascular health effects of MOF observed in humans.

## Supporting Information

Figure S1 Venn diagram showing intersections of A) differentially expressed genes and B) pathways identified in this study and in the study published by Tomé-Carneiro et al. [37].
(PPTX)

Table S1 List of differentially expressed genes following MOF consumption for 8 weeks.
(XLSX)

Table S2 Gene ontology of the differentially expressed genes.
(XLS)

Table S3 Pathways identified for differentially expressed genes using MetaCore software.
(XLS)

Table S4 Table summarizing overview of intra- and inter-individual DNA methylation variation (>10%) before/after MOF supplementation.
(DOCX)
