# Supplementary material for: Dietary Flavanols Modulate the Transcription of Genes Associated with Cardiovascular Pathology without Changes in Their DNA Methylation State
Source: PLoS One. 2014 Apr 24;9(4):e95527. doi: 10.1371/journal.pone.0095527 (PMC3998980; doi:10.1371/journal.pone.0095527)
Supplement: Figure S1 — Venn diagram showing intersections of A) differentially expressed genes and B) pathways identified in this study and in the study published by Tomé-Carneiro et al. [37]. (PPTX) [file pone.0095527.s001.pptx]

## Slide 1
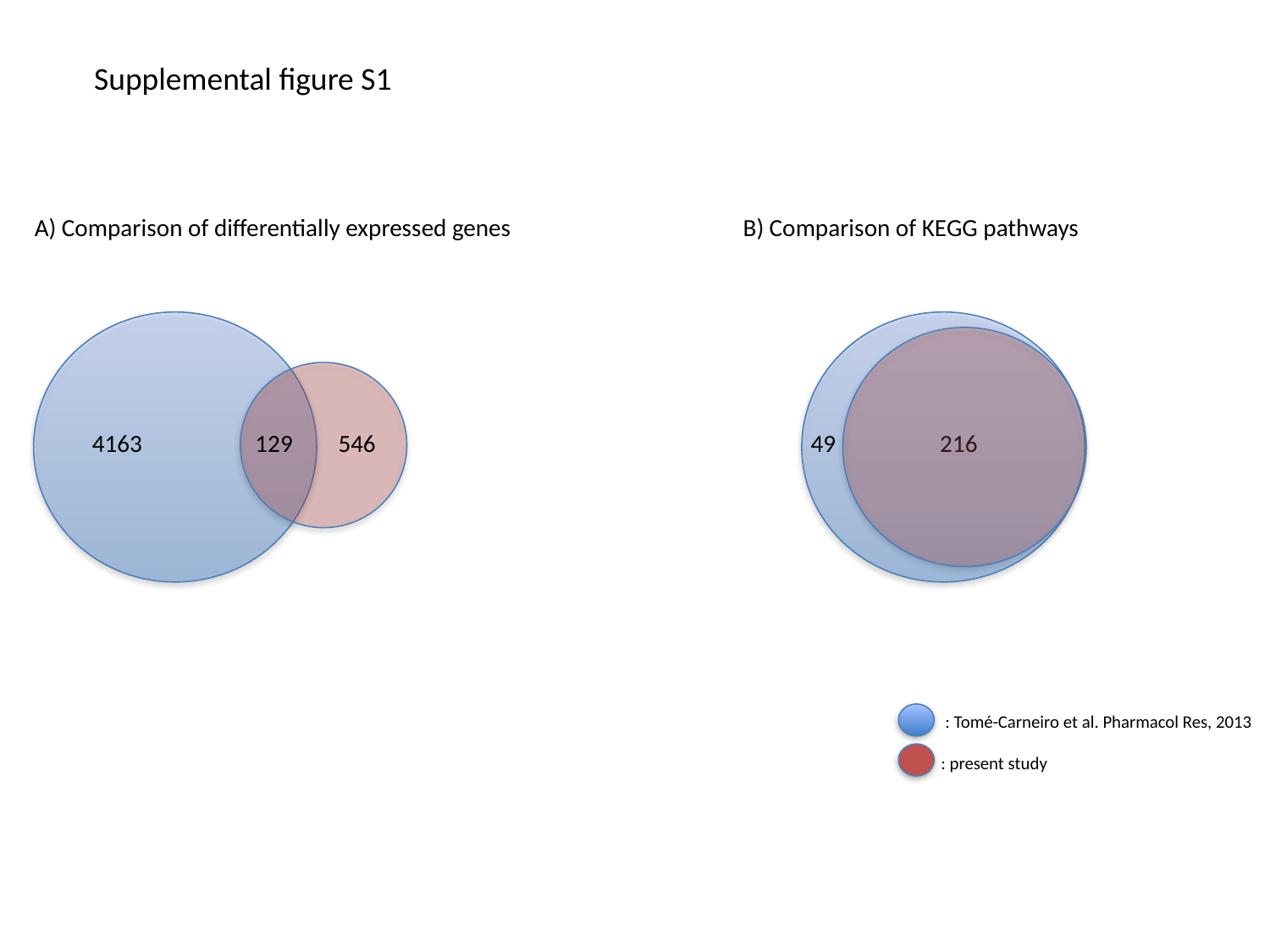

Supplemental figure S1
A) Comparison of differentially expressed genes
B) Comparison of KEGG pathways
4163
129
546
49
216
: Tomé-Carneiro et al. Pharmacol Res, 2013
: present study
