# Supplementary material for: Dietary Flavanols Modulate the Transcription of Genes Associated with Cardiovascular Pathology without Changes in Their DNA Methylation State
Source: PLoS One. 2014 Apr 24;9(4):e95527. doi: 10.1371/journal.pone.0095527 (PMC3998980; doi:10.1371/journal.pone.0095527)
Supplement: Supplementary file 2 [file pone.0095527.s005.docx]

		After MOF supplementation									
		person1	person2	person3	person4	person5	person6	person7	person8	person9	person10
Before MOF supplementation	person1	2519	15361	36828	26238	15353	22879	24660	19293	13428	12926
	person2	18131	307								
	person3	11596		1066							
	person4	9797			1460						
	person5	9556				488					
	person6	9432					14024				
	person7	11286						463			
	person8	19609							1116		
	person9	9032								118	
	person10	13742									8812

1234	**intra**-individual DNA methylation CpG probe differences (>10%) before/after MOF supplementation
1234	**inter**-individual DNA methylation CpG probe differences (>10%) before/after MOF supplementation
